# The Pathological Society of Philadelphia

**Published:** 1858-04

**Authors:** 


*The Pathological Society of Philadelphia.*—We see with great pleasure in the pages of the last number of the North American Medico-Chirurgical Review, the proceedings of this society, the founding of which has been contemplated for some time. The rich field which a city of the size of Philadelphia presents for pathological investigation, has long since demanded the organization of such a society. New York has set the example, and the great interest which its proceedings have awakened in the profession, as well as the incentive which has been given to the study of this hitherto neglected branch of science, have not failed to excite the salutary emulation of the quaker city.

The Philadelphia society was organized on the fourteenth of October, 1857, and the following officers were elected:

| | |
|---|---|
| *President*, . . . | Dr. Gross. |
| *Vice-Presidents*, . . . | Drs. La Roche and Stillé. |
| *Treasurer*, . . . | Dr. Addinell Hewson. |
| *Secretary*, . . . . | Dr. Da Costa. |
| *Assistant Secretary*, . . | Dr. T. G. Morton. |

The distinguished reputation of the officers of the society, and the talent and industry of its members, bespeak for it all the success that could be desired. The proceedings which appeared in the Jan. No. of the N. American Review, are of exceeding interest, and embody accounts of a number of valuable pathological specimens, such as fatty degeneration of the liver, cancer of the pancreas, liver, and thigh; fungus of the testicle; bony tumor of the scrotum, etc. The discussion of these specimens by men as eminent as Drs. Gross, La Roche, Stillé, and Da Costa, are useful to every one, and will form one of the most interesting features of the Review.

